# Intestinal Obstruction Due to Bochdaleck´s Hernia in an Adult

**DOI:** 10.4103/1319-3767.43280

**Published:** 2008-10

**Authors:** Miguel Echenique Elizondo, Borja Aguinagalde

**Affiliations:** Department of Surgery, Basque Country University, P. Dr. Begiristain, 105, 20014, San Sebastián, Spain. E-mail: gepecelm@sc.ehu.es

Sir,

Bochdalek's hernia is a common congenital anomaly in neonatal and postnatal patients, while being extremely rare in adults. We have examined a 36-year-old previously healthy man who presented with epigastric pain of a sudden onset that radiated to the presternal area along with nausea and vomiting. No fever or other symptoms were observed. Physical examination, laboratory data, and ECG revealed no abnormalities. A chest X-ray revealed an air bubble in the lower left hemythorax with a liquid level, whereas a flat abdominal X-ray examination showed a small bowel obstruction. Surgery was performed under general anesthesia with single lung ventilation and a laparoscopic approach. Parts of the stomach, small intestine, colon, greater omentum, and spleen were herniated into the left thoracic cavity through the posterior and lateral defect that was 8 cm in diameter. No hernia sac was identified. Herniated viscera were pulled back into the peritoneal cavity, and the defect was closed with interrupted sutures. The lung was found to expand normally in the postoperative period. The patient remained asymptomatic 24 months later.

**Figure 1:**
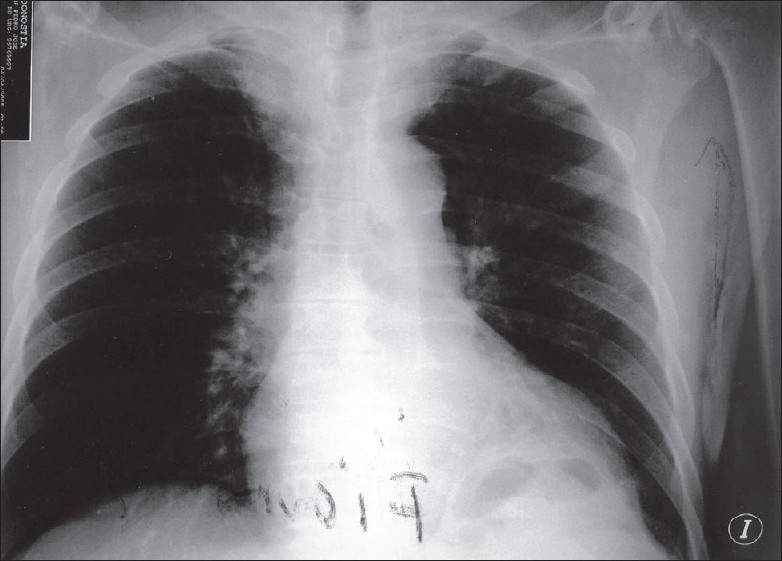
The chest X-ray on admission. PA view

In a recent study,[1] Bochdalek's hernia was diagnosed in 22 patients (17 women, five men), which represents an incidence of 0.17% based on 13 138 abdominal CT reports. The mean age of the patients was 66.6 years; none of the patients was symptomatic. In these patients, 68% of the hernias were located on the right side, 18% on the left, and 14% were bilateral. Of all hernias, 73% contained only fat or omentum, whereas 27% had solid or enteric organs including the spleen or the small or large intestine. Gale[2] showed a 2:1 frequency for right-sided cases, and a bilateral instance of 0.9%; strangulation sometimes occurs. Putative causes for late-presenting hernias include congenital herniation, blunt or penetrating trauma, physical exertion (including sexual intercourse), pregnancy, labor and delivery, sneezing or coughing, and even the ingestion of a large meal.

**Figure 2:**
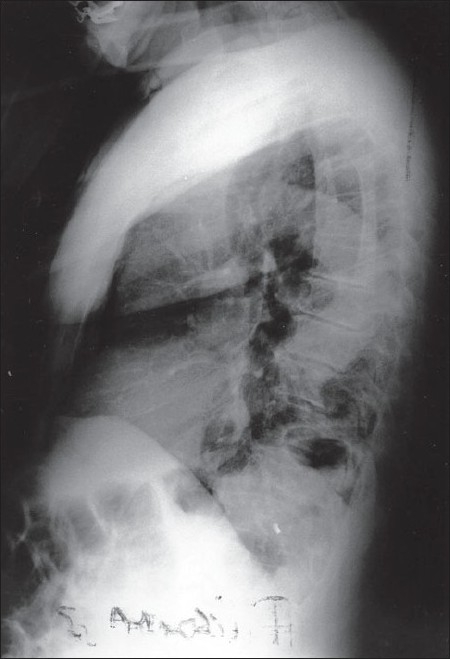
The chest X-ray on admission. L view

Diagnosis is ascertained by a combination of chest X-rays, computed tomography (CT), magnetic resonance imaging (MRI), as well as upper gastrointestinal and bowel double-contrast study. Past normal chest X-rays do not exclude the presence of Bochdalek's hernia.

For right-sided Bochdalek hernias, a transthoracic or thoraco-abdominal approach is preferable. For left-sided hernias, some advocate a transthoracic approach, while others suggest a transperitoneal one. A transthoracic approach enables direct observation of the herniated viscera. A transperitoneal approach also allows the surgeon to confirm the position of the viscera after “pull back” and repair any malrotation, if present.

**Figure 3:**
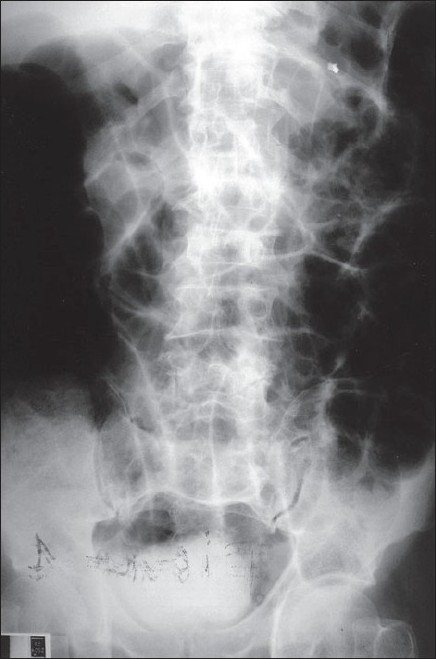
Flat abdominal X-ray. Intestinal obstruction

Minimally invasive procedures—both by thoracic or abdominal approaches—have been demonstrated to be effective.[3,4] In summary, Bochdalek's hernias are likely to be more common in the general population than previously reported.
